# American Academy of Dental Science

**Published:** 1890-03

**Authors:** 

**Affiliations:** American Academy Dental Science


### Reports of Society Meetings.

### AMERICAN ACADEMY OF DENTAL SCIENCE.

(Continued from February number.)

*President Seabury.*—The next matter in order is a paper entitled "Rapid Method of Inserting and Finishing Contour Fillings without the Aid of a Matrix," by Dr. Joseph E. Waitt, Boston.

*Dr. Waitt.*—Gentlemen of the American Academy of Dental Science, the method that I present to you is not new, neither is it entirely my own; it is a chip from here and a chip from there, and putting them all together, I reached the result of which I will show you a specimen, and tell you how I accomplished it. (For paper, see page 136.)

*Dr. Andrews.*—The subject is so interesting that I wish we had a black-board here, so that Dr. Waitt might demonstrate his method more clearly; and I move you, sir, that we have one hereafter for the use of the members.

*Dr. Ames.*—I should like to ask Dr. Waitt how long it would take to fill a cavity of the size shown in specimen No. 2. Could you do it in half an hour?

*Dr. Waitt.*—You could finish the filling in about half an hour. The filling, as far as you see it there, or with one more layer, can be done in about ten or twelve minutes. It will take as long to build that top as it does the three-quarters or seven-eighths of the filling up to that point.

In making these squared pellets, or squared cylinders, I take these ropes, if I want to make a very long cylinder, No. ½, and fold this rope on itself lengthwise, then over and over. If I want to make a large cylinder, I start with full width of rope and fold it on itself and roll it somewhat larger than the cylinder is to be when squared.

*Dr. Barker.*—So far as I was able to comprehend the doctor, the method he describes is not dissimilar to the one I have been using for several years, and doubtless many others employ it. In regard to the preparation of the cavity, he made a remark that attracted my attention. It was this: "There are no retaining pits used." I apprehend that by far the larger portion of good operators have practically abandoned retaining pits.

The preparation of the doctor's cavity is almost identical with the method that is described in nearly all the standard works on operative dentistry. Without the use of a diagram, or a black-board, I confess my inability to grasp the minutiæ of the locking and interlocking he describes.

The method consists simply, as I understand it, in laying a mat, a flattened cylinder, or a block of unannealed foil on the floor of the cavity and condensing it. The floor may be thus formed of one block, if it be sufficiently large; place now a flattened cylinder at each border, condensing them laterally against the walls; lay on the bottom one large or three smaller blocks, which, when condensed, expand laterally, thus fastening the side-pieces in position; more blocks may be introduced at the bottom and be treated in the same way. When the cavity is two-thirds or more full, begin to work on cohesive foil. It will readily cohere by the joint effects of molecular and mechanical cohesion, if good serrations are employed; complete the filling with cohesive foil. The burnisher should be employed on the proximal aspect of the unannealed foil during the various steps of the operation.

*Dr. E. G. Tucker.*—Mr. President and Fellows, I have never used gold foil in any other form but pellets of different sizes, the small, delicate ones in front teeth, large ones in large cavities (grinding surfaces), where great *packing* pressure could be given. By carrying out the filling to the edges with these small pellets and *sharp-pointed* pluggers, you can get as perfect a filling—polished and durable—with No. 4 soft gold and hand pressure as can be made with automatic mallet and hard gold. I have tested this mode of filling fifty-four years, and have used no other to the present day. I will show the Academy four front teeth that I filled for a young man in Harvard College, which were used thirty-six years after being filled, and then were extracted on account of general disease of the sockets of all his teeth, from the effects of which they became loosened. As you will see, the fillings are still in excellent condition and preserved those teeth during that long period.

*Dr. Williams.*—In regard to mats, or pellets, as they used to be called in Dr. Tucker's early days, this method reminds me of the way his brother and he used to make fillings that made such a reputation for American dentists. I think it was similar to the manner described by Dr. Waitt.

*Dr. Briggs.*—I do not perfectly understand Dr. Waitt's paper. I wish I could have it explained, as Dr. Andrews suggests, with a black-board. I think he has given us something new, whereas I am quite sure that Dr. Barker has given us something old. His is the ordinary soft filling; but I think that Dr. Waitt's method may have something more in the finishing, perhaps, than any mere question of using soft or cohesive gold. I do not know, perhaps some of the members understand it better than I do, but it seems to me that the other methods spoken of are very old, and I hope that Dr. Waitt will endeavor to explain his process more fully by the use of a diagram.

*Dr. Andrews.*—I want to say a few words criticising the shape of the cavity formed by Dr. Waitt. We understand that the shape of the cavity is exaggerated, but you will find the cervical wall to be the weakest part of the whole cavity. We know that if we cut across the tubuli, at the cervical wall, there is dead tissue there, and I should think that the filling could be quite as well made if the floor of the cavity was cut square across or made to slant outward rather than inward.

*Dr. Waitt.*—I said in the first place that it was an exaggerated case. I expect that each one will use his own judgment. The filling can be practically put in as well on a flat floor as it can on a floor that has a groove cut across it, but where the tissue is comparatively hard, a slight groove can be made. I use the finest inverted cone that the S. S. White Company makes. The filling is a soft gold filling, with the exception that a layer of cohesive gold is placed between the layers of soft foil. The contour is made entirely by burnishing. The pellets are put into the cavity with the ends standing out, somewhat longer than the cavity is deep. It is the length of these out-standing ends that makes the contour. Then again, where each layer is locked, that makes it a little stronger, and you have a hard edge to burnish on to.

*Dr. H. A. Baker.*—I thought that when Dr. Andrews began to speak he was going to bring up just what I had in my mind, only my exceptions are at the other end of the cavity. What I wish to call attention to is the way in which the essayist forms his grooves. It seems to me that if he brings his grooves clear up through the enamel of the crown, he leaves a sharp corner which necessarily must be very weak, and in my patients' mouths that corner would break off just as sure as the world. To avoid this, I

should bring the groove up under the enamel and not cut through it, and leave the corner on a bevel, and protect it by a covering of gold, which would resist the occlusion. Another point in regard to his cylinders: You make a cylinder of rope, and one gets into an elliptical shape, and you cannot avoid it; the result is, your cylinder is thicker in the middle than it is at the ends. Now, in the place of ropes, I would take non-cohesive pellets and flatten them out, lay the flat sides one upon another until you get the desired thickness; then, bending the ends towards each other, the result is, one gets a cylinder in the cavity like so many leaves in a book, one upon another, with one end against the wall of the cavity, the other against the matrix; and thus you have an even thickness throughout. This should fill the cavity about one-third full, then forming an anchorage above this, and finishing out with cohesive gold, protecting your corners as I described before, and you will have a filling which will stand the test of severe usage.

*Dr. Briggs.*—As I understand it, it is not so much the shape of the cavity as it is the filling. It may be, it is boring the others, but I wish that Dr. Waitt would give us a *résumé* of his process of putting in the filling.

*Dr. Waitt.*—Perhaps you are getting too much, gentlemen. I told you in the first place that it was not entirely my own, but a chip from here and a chip from there, and putting these together I got this out of it, and this I can do. Now, the preparation of the cavity or the making of the cylinder is immaterial. I do know that I can take a patient in my chair and fill a cavity in one-quarter of the time that I can do it with cohesive gold, or any other method that I have ever seen. It is my way, and yet it is not my way entirely, because it is a combination of the parts of several other methods.

In the first place, if we look at the end of the cavity from front to back, we see here these squares laid side by side. First, place these squares across the bottom and only partially condense them with a large plugger. Cohesive gold is then placed over this in shreds, as it were, of the thickness of one single sheet at a time, and with a plugger, the serrations of which are sharper than for ordinary condensing, make a thorough mechanical union with the soft gold. I put on perhaps three or four thicknesses of this single foil and carry them right straight down, as hard as I can condense it by hand pressure. Now, I do not condense beyond what is to be the contour of the tooth, for by so doing you are likely to push the plugger through that portion. After the first layer is put in, locked, and condensed, I take a burnisher and carry it up and about the extending ends and form the contour, and burnish away the small amount of overlapping gold. That layer is then practically finished. I claim that after that layer has been put in, you could leave it for weeks, and then take out the filling, and the bottom of your cavity will be perfectly dry.

*Dr. Clapp.*—I would like to ask Dr. Waitt if he uses a mallet at all?

*Dr. Waitt.*—When I get clear up to the surface, which would become the grinding edge, I then use a mallet. I have seen soft gold fillings put in by hand-pressure where the patient has ground and articulated on the filling, and it was not broken. I cannot do it. I would be very happy to learn how to condense soft gold by hand-pressure to stand such a strain.

*Dr. Andrews.*—May I ask Dr. Waitt the object of the cohesive gold?

*Dr. Waitt.*—The cohesive gold, as you see it in this layer, extends clear out to what would be the edge of the contour of the tooth. It acts as a face or a resistance to burnish against.

Subject passed.

*President Seabury.*—Dr. F. G. Eddy will present a new mouth-piece for saliva-ejectors.

*Dr. Eddy.*—There is a mistake in the announcement on the card that I was to present a *new* mouth-piece. I do not know how it happened. There is nothing new in regard to this mouth-piece. It is simply the old mouth-piece with lengthened shank, and with a stop-cock put a little lower down, so as to be under the control of the patient.

As I was leaving the office to-day, Dr. G. W. Porter, a surgeon of Providence, handed me this box, part of the contents of a dermoid tumor, removed a few days before, and within that, among some hair and some fatty substances, he found these teeth, and also these pieces of bone. Out of sixty ovarian tumors he has found but twelve dermoid cysts. This is the only one in which he has found any teeth. With them he also found the left half of a lower maxillary bone.

*Dr. Andrews.*—The little stop-cock in Dr. Eddy's ejector is certainly an improvement, but I had hoped that we were going to get something that would not suck in the soft tissues.

*President Seabury.*—I have an old ejector, the holes of which are on top.

*Dr. Briggs.*—I like this ejector very much. I would like to ask Dr. Eddy if, in using it in ordinary operations, *débris* would be likely to get into the cracks and stop them up very easily?

*Dr. Eddy.*—I think not. I use it daily.

*Dr. Waitt.*—I think the best mouth-piece I ever saw was one shaped something similar to this, but the end of the tube came down nearly to the bottom of a little cylinder made of perforated metal and surrounding it. The saliva can come up in it three-fourths of its length, then pass down and go into the tube. I have used one of them five years, and I never had any trouble with it. I would like to ask Dr. Eddy, Will these fine slits let thick, stringy saliva pass through them?

*Dr. Eddy.*—Yes, sir.

*Dr. Williams.*—I have an impression that this plan of Dr. Eddy's, of having slits instead of holes, will be an improvement. There is a chance for a slit to clear itself, while a hole blocks up. There is an objection to the weight of this ejector. I have a short one, not more than five inches at the most. I put in the lightest rubber tubing that I can find that will not collapse by suction, so as to avoid any sense of weight in the mouth. I find the patients feel relieved, and consider it somewhat of a luxury.

*Dr. Andrews.*—One of the advantages of this saliva-ejector is that the patient can hold it in the mouth; but what I wanted to suggest was that the mouth-piece might be difficult to cleanse. Any one of you who has tried to clean a saliva-ejector must have been surprised at the amount of filth that can get into an instrument of this kind.

*Dr. Codman.*—I have noticed among practitioners a growing tendency to make dental operations complicated. I think that has been so very largely in the use of the saliva-pump. I think, in a great many cases, it is entirely unnecessary, and should be dispensed with. The drawing out of a person's system of a pint to a quart of saliva must certainly be very exhausting, and this is done in many cases where the saliva-pump is not needed at all. I don't think I have used one in six months, for I find by a proper adjustment of the rubber dam with ligatures my patients get along much easier than they would by the use of the saliva-pump.

*Dr. Meriam.*—I should like to present, Mr. President, as chairman of the Executive Committee of the Massachusetts Dental Society, a cordial invitation to the Academy to our next meeting, to be held on Thursday next. In addition to our usual programme we shall have a small exhibit, not as extensive as last summer. The annual address will be delivered by Rev. Alexander McKenzie, D.D., secretary of the Board of Overseers of Harvard University. The address will be given at 4.30 o'clock in the afternoon, and we hope there will be a large attendance. It will be given in the large hall of the Young Men's Christian Association. We have sent an invitation to the faculty and students of the Harvard Dental School, and also to the faculty and students of the Boston Dental College, and we trust that the attendance will be large, to do justice to our orator.

*Dr. Fillebrown.*—Mr. President, I move the invitation be accepted and placed on file by this society.

Dr. Waitt exhibited a nitrous oxide light for photo-microscopic purposes with the following description:

The principle is old in that it is a combination of the Knapp blow-pipe simply adapted to a burner which carries a piece of lime. It is after the principle of the oxyhydrogen light, with the exception that we use ordinary illuminating gas and nitrous oxide gas. By this I get, as you see, a very powerful white light. In my first experiments it would spit and sputter, but I finally found that the trouble was in getting the gas out of the cylinder. It would persist in freezing. If I blow off about twenty-five gallons, or use a seventy-five- or fifty-gallon cylinder, I get the result which you see here. A light, as Dr. Andrews knows, for photographic purposes, needs to be an intense white light. This light can be used for the projection of pictures on a screen, and for a picture up to twelve or fifteen feet in diameter it is perfect. In a course of lectures by Dr. Dearborn I illuminated a circle of twelve feet diameter, and he said the pictures were fully as well illuminated as if they were shown by his regular operator with the oxyhydrogen light. The plates were the ordinary three and one-fourth by four.

In the illuminating of stereopticon pictures all that is required is a bright spot or line of light; in other words, the whole thing to be reached is to get the most intense white light into the smallest possible spot. After I once get this light in this way, and the cylinder of gas down to where it will blow readily, we can regulate the light by a stop-cock in this cylinder.

William H. Potter, D.M.D.,
*Editor American Academy Dental Science.*

